# Workshop Safety Helmet Wearing Detection Model Based on SCM-YOLO

**DOI:** 10.3390/s22176702

**Published:** 2022-09-05

**Authors:** Bin Zhang, Chuan-Feng Sun, Shu-Qi Fang, Ye-Hai Zhao, Song Su

**Affiliations:** 1 School of Electronic and Automation, Guilin University of Electronic Technology, Guilin 541004, China; 2 Liuzhou Wuling Automobile Industry Co., Ltd., Liuzhou 545000, China

**Keywords:** YOLOv4-tiny, safety helmet wearing detection, convolutional block attention module, label smoothing, spatial pyramid pooling structure, K-Means++ clustering algorithm

## Abstract

In order to overcome the problems of object detection in complex scenes based on the YOLOv4-tiny algorithm, such as insufficient feature extraction, low accuracy, and low recall rate, an improved YOLOv4-tiny safety helmet-wearing detection algorithm SCM-YOLO is proposed. Firstly, the Spatial Pyramid Pooling (SPP) structure is added after the backbone network of the YOLOv4-tiny model to improve its adaptability of different scale features and increase its effective features extraction capability. Secondly, Convolutional Block Attention Module (CBAM), Mish activation function, K-Means++ clustering algorithm, label smoothing, and Mosaic data enhancement are introduced to improve the detection accuracy of small objects while ensuring the detection speed. After a large number of experiments, the proposed SCM-YOLO algorithm achieves a mAP of 93.19%, which is 4.76% higher than the YOLOv4-tiny algorithm. Its inference speed reaches 22.9FPS (GeForce GTX 1050Ti), which meets the needs of the real-time and accurate detection of safety helmets in complex scenes.

## 1. Introduction

Wearing a safety helmet is an important safety protection measure for a construction site and manufacturing shop. The detection of safety helmet wearing is one of the important measures for enterprise safety management. With the development of computer vision [1], the target detection algorithm in deep learning has been widely applied to the safety helmet wearing detection of workers in the manufacturing shop [2,3,4,5,6], which can realize unmanned and low-cost detection. This greatly ensures the production safety of the workshop and improves production efficiency, which greatly illustrates the practical application value of the target detection algorithm in the production field.

In recent years, many scholars at home and abroad have proposed the safety helmet detection method based on deep learning. Cheng et al. [7] proposed a SAS-YOLOv3-tiny algorithm, constructed a light Sandglass Residual (SR) module based on depth-wise separable convolution and the channel attention mechanism. The spatial pyramid pooling (SPP) module was improved to merge into the backbone network, and the final mAP could reach 80.3%. Nan et al. [8] proposed a safety helmet detection dynamic model based on the critical area attention mechanism. The model firstly detects human objects in the image, locks the human head area through the critical area attention mechanism, and finally, achieves safety helmet detection through multiple up-samplings to highlight the helmet feature information, and the mAP can reach 92.68%. Zhou et al. [9] proposed an object detection algorithm based on CenterNet, which uses a U-shaped feature pyramid to fuse multilayer features. On the basis of the feature pyramid structure, a global guidance module and a feature integration module are added to improve the sensitivity to small-scale objects and, finally, achieve a score of 87.8% mAP. Xiao et al. [10] proposed a fast detection algorithm for helmet wearing based on improved SSD by replacing the backbone network VGG-16 in SSD with a lightweight convolutional neural network MobileNetV3-small. This reduces the parameters of the model and uses the feature pyramid structure to fuse the deep features with the shallow features, which improves the detection accuracy. The AP of the model can reach 91.1%. Although these methods have achieved good results, due to the complex detection background, object occlusion, and dense objects, problems such as poor detection stability, low detection speed, and inaccurate safety helmet detection will result.

Additionally, many researchers have also developed the safety helmet detection model based on the YOLO series of algorithms. Among them, Deng et al. [11] modified the basis of the YOLOv4 model by applying the K-Means algorithm to cluster the dataset in order to obtain a more targeted a priori frame for prediction. A multi-scale training strategy is then adopted to improve the adaptability of the model to different detection scales. The mAP and detection speed of this model reaches 92.89% and 15 f/s, respectively. Zeng et al. [12] optimized the accuracy and speed of the model by replacing the cumbersome overlap of multiple convolutional modules in the YOLOv4 feature pyramid with cross-stage hierarchical modules. At the same time, the output of the YOLOv4 feature layer and the linear transformation of the anchor points are changed to improve the performance of YOLOv4 in detecting small objects. The model achieved a mAP and detection speed model of 93.37% and 29 f/s, respectively. Gao et al. [13] replaced the backbone network of YOLOv4 with the MobileNetV3 network. A depth-wise separable convolution is adopted to reduce the amount of parameters in the backbone network, and H-swish is utilized to improve the performance of the model. The mAP of this model reaches 98.2%, and the detection speed reaches 40 f/s. The detection methods used by the above scholars have achieved remarkable results, but these network models are too complicated, and experimental reproduction requires higher performance hardware equipment. Considering that the model required for practical application should meet the advantages of real-time and accuracy of workshop detection, this paper used YOLOv4-tiny with fewer parameters as the basic model for improvement and optimization.

The rest of the paper is organized as follows. Section 2 will explain the principles and problems of the original algorithm of YOLOv4-tiny. Section 3 will describe the innovation points of the improved algorithm (SCM-YOLO) in detail. Section 4 will show some experimental results and analyze them. Finally, in Section 5, this paper will be summarized, and some future works will be proposed.

## 2. YOLOv4-Tiny Object Detection Algorithm Model

Compared with the two-stage detection algorithms such as Faster R-CNN [14] and Mask R-CNN [15], the one-stage object detection algorithm YOLOv4-tiny with faster detection speed is more in line with the actual application requirements, meeting the high real-time requirements of safety helmet wearing detection tasks in the production workshop. Therefore, this paper used YOLOv4-tiny as the basic network for improvement and optimization. The improved SCM-YOLO algorithm has a higher detection rate for small objects and occluded objects. Its accuracy and real-time performance are more suitable for application requirements.

### 2.1. YOLOv4-Tiny Model Structure

**Figure 1:**
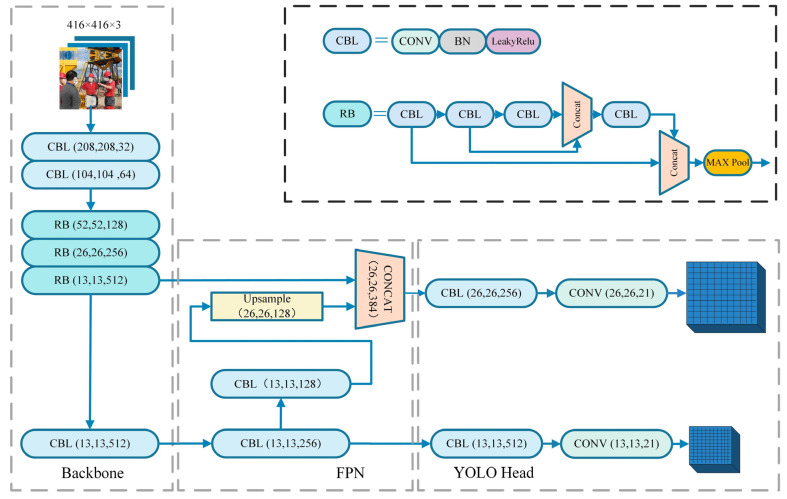
YOLOv4-tiny network structure.

YOLOv4-tiny is a lightweight model proposed after YOLOv4 [16]. The pruning operation is performed on the basis of the YOLOv4 model [17], which greatly reduces the amount of network parameters and improves the detection speed. YOLOv4-tiny consists of three parts, namely the backbone extraction network, the Feature Pyramid Network (FPN) [18], and the detection network Head. The YOLOv4-tiny network structure diagram is shown in Figure 1.

**Figure 2:**
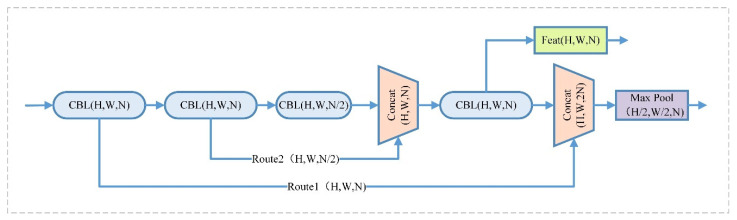
CSPNet structure.

CSPDarknet53-tiny of the backbone network consists of 3 CBL (DarknetConv2D_BN_Leaky) modules and 3 RB (Resblock_body) modules. The CBL module contains a 2D convolutional layer, a batch normalization layer, and a LeakyReLU activation function. The CSPNet (Cross Stage Partial Network) is introduced into the RB module, so that the network structure can achieve richer gradient combination information while reducing the amount of computation. First of all, the pictures passed into the network will be resized into a unified format of 416 × 416 × 3 in size. Through two convolution operations with a convolution step size of 2, normalization, and activation function processing, a 104 × 104 × 64 feature map is obtained. Then, the feature map is passed into the CSPNet structure. The residual block in the CSPNet structure is divided into two parts [19]. Among them, the residual edge part is directly connected to the end, and the backbone part continues to stack the residual blocks, as shown in Figure 2, where H, W, and N represent the height, width, and channel number of the feature map, respectively.

The backbone network outputs a total of two effective feature layers, of which Feat1 is output through the CSPNet structure in the third RB module, and Feat2 is the final output of the backbone network. These two effective feature layers are passed into the FPN. In the FPN, one convolution and CBL operation will be performed on Feat2, followed by an up-sampling operation, and then stacked and convolved with Feat1. In this way, the feature layer with high semantic information and the feature layer with low semantic information are fused to improve the detection accuracy of the network model.

In the Head part, the decoding operation is performed on the obtained feature map. YOLOv4-tiny has 3 prior boxes of different sizes for each grid of each feature map, so the final number of channels is 3 × (1 + 4 + N). Among them, 1 is whether the object is included in the prior frame, 4 is the position information of the prior frame, and N is the number of classifications. Finally, the corresponding predicted bounding box is obtained by the prediction confidence, and then, the non-maximum suppression algorithm is used to remove the redundant bounding box to obtain the final detection box of the model.

### 2.2. Problems of YOLOv4-Tiny Algorithm

As a lightweight network model, YOLOv4-tiny has a significantly better detection speed than other object detection algorithms such as SSD [20] and Faster R-CNN, which can meet the application requirements of real-time detection. However, in terms of detection accuracy, it still needs to be improved in practical production with complex backgrounds.

Although the CSPDarknet53-tiny structure in the YOLOv4-tiny algorithm greatly simplifies the complexity of the network structure and improves the algorithm accuracy, it also reduces the detection accuracy. The activation function LeakyRelu in CSPDarknet53-tiny leads to slower convergence due to the non-zero mean output. Due to the different interval functions, the output results are also inconsistent; it cannot provide consistent relationship prediction for positive and negative input values.

The size of the feature map extracted by the backbone network needs to be fixed when entering the FPN structure. However, conventional clipping, stretching, and other operations will cause distortion of the feature map. The subsequent 1 × 1 and 3 × 3 convolution operations will also lead to the problem of incomplete feature information extraction due to the small receptive field, which will reduce the detection accuracy of the model and cause the problem of missed detection in safety helmet detection.

## 3. SCM-YOLO Detection Model Based on Improved YOLOv4-Tiny

### 3.1. SCM-YOLO Model Structure

**Figure 3:**
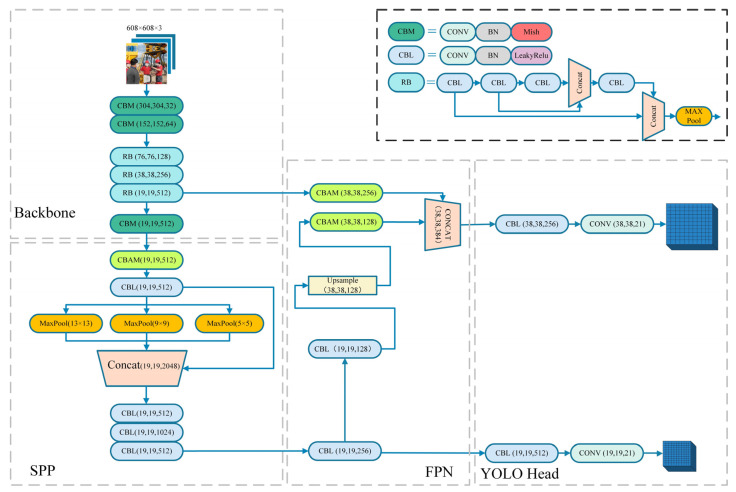
SCM-YOLO network structure.

In order to make the model adapted to the complex scene of the workshop and improve the detection accuracy, we first changed the activation function of the backbone network to the Mish activation function. Secondly, the Convolutional Block Attention Module (CBAM) and the Spatial Pyramid Pooling (SPP) structure were introduced. Finally, in the training process and the data preprocessing stage, label smoothing strategy, K-Means++ clustering algorithm, and Mosaic data enhancement were introduced to improve the robustness and accuracy of the model. The framework of the real-time detection model for safety helmet wearing based on SCM-YOLO is shown in Figure 3.

### 3.2. Improvement of Backbone Network Activation Function

**Figure 4:**
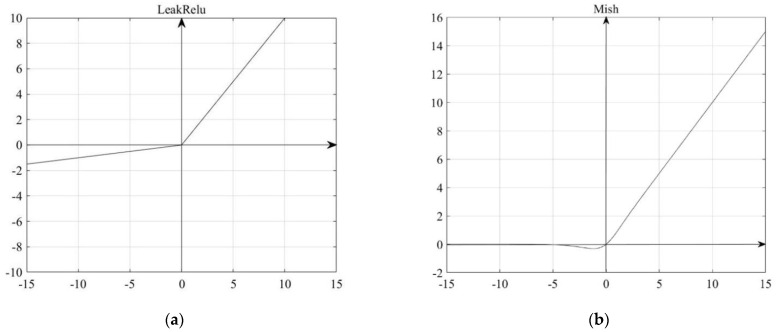
Comparison of the activation functions.

In the YOLOv4-tiny algorithm, the nonlinear activation function used by the backbone network is LeakyRelu. Compared with the LeakyRelu activation function, the nonmonotonicity of the Mish activation function makes the critical points in the positive and negative intervals not completely truncated but transition through a small negative gradient. Reference [21] contributes to stabilizing the network gradient flow and ensuring the flow of information. In addition, the lower bound of the Mish activation function enables the parameter amplitude to be controlled so as not to make the model too complex, which helps to achieve a strong regularization effect. The activation function comparison is shown in Figure 4.

LeakyRelu activation function [22] expression is shown in Formula (1):

(1) $$LeakyRelu=\{\begin{array}{ll} z & z>0\\ \alpha z & z\leq 0,\alpha =0.1 \end{array}$$

The back-propagation derivation process is: setting the output of layer l as $z^{l}$, the output after LeakyRelu is $z^{l+1}$. Noting that the partial derivative of the loss function L with respect to the output $z^{l}$ of the lth layer as $\delta^{l}=\partial L/\partial z^{l}$, then the partial derivative of the loss function L with respect to the lth layer is shown in Formula (2):

(2) $$\begin{array}{ll} \delta^{l} & =\frac{\partial L}{\partial z^{l+1}}\frac{\partial z^{l+1}}{\partial z^{l}}\\ & =\delta^{l+1}\frac{\partial LeakyRelu(z^{l})}{\partial z^{l}}\\ & =\delta^{l+1}\left\{ \begin{array}{ll} 1 & z^{l}>0\\ \alpha & z^{l}\leq 0,\alpha =0.1 \end{array}\right.\\ & =\left\{ \begin{array}{ll} \delta^{l+1} & z^{l}>0\\ \alpha \delta^{l+1} & z^{l}\leq 0,\alpha =0.1 \end{array}\right. \end{array}$$

The Mish function activation function expression [23] is shown in Formula (3):

(3) $$Mish(x)=x*\tanh(\ln(1+e^{x}))$$

where $\ln(1+e^{x})$ is the expression of soft-plus activation function. The back-propagation derivation process is: set the output of the l layer as $x^{l}$, and the output after Mish is $x^{l+1}$. Then, the partial derivative of the loss function L with respect to the lth layer is shown in Formula (4):

(4) $$\begin{array}{ll} \delta^{l} & =\frac{\partial L}{\partial x^{l+1}}\frac{\partial x^{l+1}}{\partial x^{l}}\\ & =\delta^{l+1}\frac{\partial Mish(x^{l})}{\partial x^{l}}\\ & =\delta^{l+1}\frac{e^{x}\omega }{\varphi^{2}} \end{array}$$

Among them, $\omega =4(x+1)+4e^{2x}+e^{3x}+e^{x}(4x+6),\varphi =2e^{x}+e^{2x}+2$.

### 3.3. SPP-Spatial Pyramid Pooling Structure

**Figure 5:**
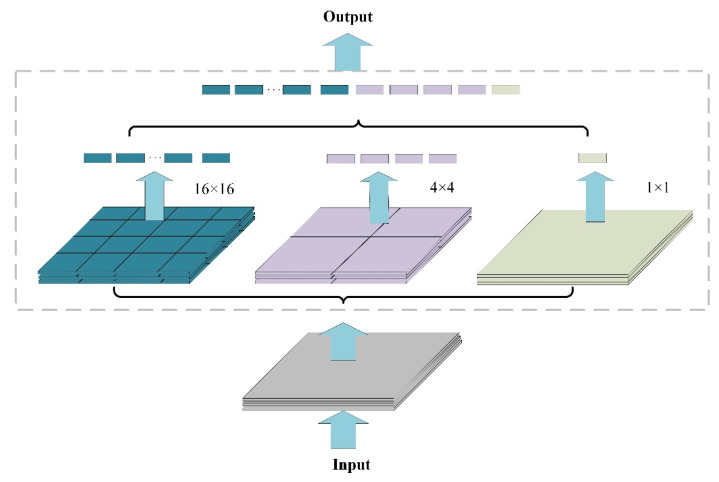
The original SPP structure.

In the convolutional neural network, the fully connected layer has a fixed size requirement for the input feature map. However, during normal crop and stretch operations, the size and aspect ratio of the input image will be compressed and changed. This will cause the distortion of the input feature map, thereby losing some effective feature information and reducing the detection accuracy of the model. The SPP [24] structure proposed by He et al. can solve this problem very well. Features are extracted and pooled at different scales of the same feature map by using multilevel size space windows. In this way, the size of the input feature map is fixed, the effective information of the feature map extracted by the backbone network is preserved, and the detection accuracy of the algorithm is optimized. The structure is shown in Figure 5.

**Figure 6:**
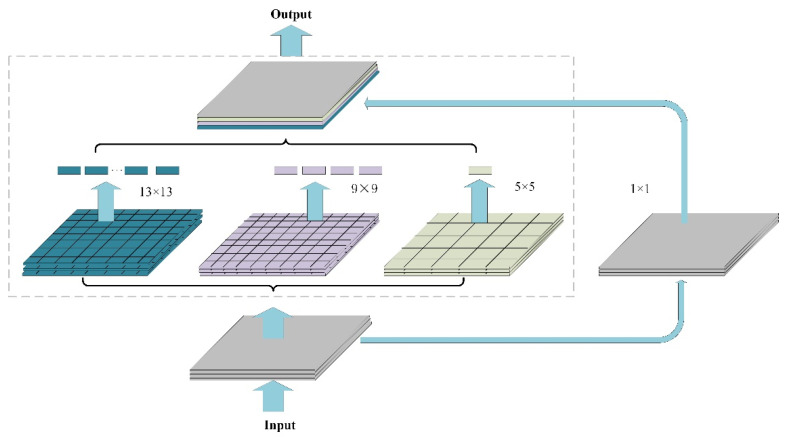
SPP structure in this paper.

Inspired by the SPP structure, this paper performed the SPP pooling operation on the output of the backbone network, as shown in Figure 6. In specific operations, pooling operations based on different separate-block are applied to the input feature map, in which the pooling operation module consisted of three maximum pooling layers; the pooling window sizes were 13 × 13, 9 × 9, and 5 × 5; and the stride was 1. To ensure that the final feature dimensions were consistent, each pooling window extracted one feature as a dimension. The original global feature information without the pooling operation was fused with the local feature information with different granularities after the pooling operation. In this way, the multiscale fusion of local features and global features was realized, which improved the receptive field of the model and also enhanced the ability of the feature layer to express the object, thereby improving the detection accuracy of the model.

### 3.4. CBAM-Convolutional Block Attention Module

Since the detection accuracy of YOLOv4-tiny for small objects still has room for improvement, this paper introduced the CBAM module to make the network focus on the region of interest and removed the feature redundancy in the channel and space of the feature map to improve the detection accuracy of small objects.

**Figure 7:**
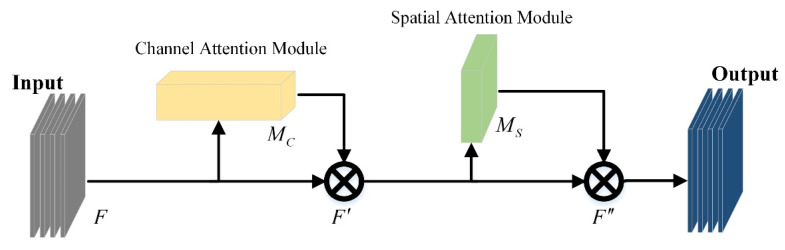
CBAM structure.

The attention mechanism is a signal processing mechanism imitating the human brain, and this strategy has good adaptability and gain for computer vision tasks [25]. The CBAM module also obtains the importance level of pixels through supervised learning [26] and assigns information weights in the channel and space dimensions, as shown in Figure 7.

The CBAM module sequentially infers a channel attention map $M_{C}$ with a size of 1×1×C and a spatial attention map $M_{S}$ with a size of H×W×1, and its expression is shown in Formula (5):

(5) $$\begin{array}{l} F^{\prime }=M_{C}(F)⊗F\\ F^{\prime \prime }=M_{S}(F^{\prime })⊗F^{\prime } \end{array}$$

where ⨂ denotes element-wise multiplication, and $F^{\prime }$ and $F^{\prime \prime }$ are the outputs after the channel attention module and the spatial attention module.

In the channel attention module, the maximum pooling operation and the average pooling operation are first used for the input feature map to compress its spatial dimensions and remove redundant features [27]. The processed feature map is sent to a Multi-Layer Perceptron (MLP) for feature extraction. Then, the feature weight information output by MLP is summed and sigmoid activated to generate the final channel attention map $M_{C}$. The obtained result $M_{C}(F)$ is shown in Formula (6).

(6) $$\begin{array}{ll} M_{C}(F) & =\sigma (MLP(AvgPool(F))+MLP(MaxPool(F)))\\ & =\sigma (W_{1}(W_{0}(F_{avg}^{C}))+W_{1}(W_{0}(F_{max}^{C}))) \end{array}$$

**Figure 8:**
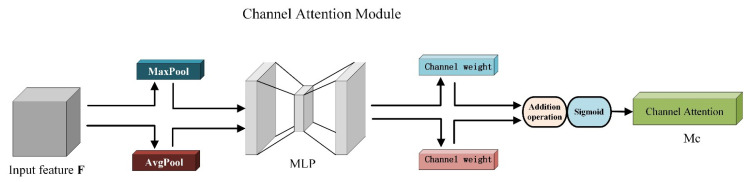
Channel attention module structure.

Among them, σ represents the sigmoid function, and $W_{0}$ and $W_{1}$ represent the nonlinear feature changes performed by the two fully connected layers. $F_{avg}^{c}$ and $F_{max}^{c}$ represent the average pooling operation and the maximum pooling operation, respectively, and the structure is shown in Figure 8.

**Figure 9:**
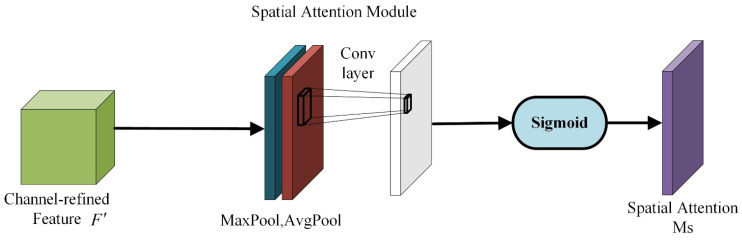
Spatial attention module structure.

In the spatial attention module [28], the maximum pooling operation and the average pooling operation are sequentially performed on the input feature map, and the compressed result is input into the application convolution layer for convolution operations. Finally, after the sigmoid activation function, a spatial attention map $M_{s}(F)$ of size R×H×W is generated. $M_{s}(F)$ is shown in Formula (7):

(7) $$\begin{array}{ll} M_{S}(F) & =\sigma (f^{7\times 7}(\left[AvgPool(F);MaxPool(F)\right]))\\ & =\sigma (f^{7\times 7}(\left[F_{avg}^{S};F_{max}^{S}\right])) \end{array}$$

where σ represents the sigmoid function, and $f^{7\times 7}$ represents a convolution operation of size 7 × 7. $F_{avg}^{s}$ and $F_{\max}^{s}$ represent the average pooling operation and the maximum pooling operation, respectively, and the structure is shown in Figure 9.

### 3.5. Improvement of Model Training Process

#### 3.5.1. Label Smoothing Regularization

In the sample training of YOLOv4-tiny, the one-hot label is used to calculate the cross-entropy [29], which is prone to overfitting when the amount of data is small. To address this issue, this study added a label smoothing regularization strategy [30] to the training process. Noise was introduced by softening the distribution labels of real samples, which weakened the class weights of real sample labels when calculating the loss function, thereby improving the generalization ability of the network. The cross-entropy function expression is shown in Formula (8):

(8) $$loss=-\sum_{i=1}^{K}q_{i}log(p_{i})$$

where p represents the predicted probability, and q represents the true probability. The mathematical expressions of p and q are shown in Formulas (9) and (10), respectively:

(9) $$p_{i}=\frac{exp(z_{i})}{\sum_{j=1}^{k}exp(z_{j})}$$

(10) $$q_{i}=\{\begin{array}{cc} 1 & i=y\\ 0 & i\neq y \end{array}$$

where z represents the output value of the corresponding category of the current sample.

The real probability distribution after adding the label smoothing regularization strategy is shown in Formula (11):

(11) $$q_{i}=\left\{ \begin{array}{ll} 1-\varepsilon & i=y\\ \varepsilon /(K-1) & i\neq y \end{array}\right.$$

where K represents the number of classes, and ε represents a small noise constant.

#### 3.5.2. Mosaic Data Enhancement

**Figure 10:**
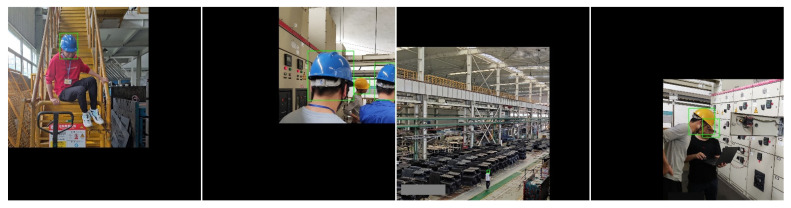
Image preprocessing.

**Figure 11:**
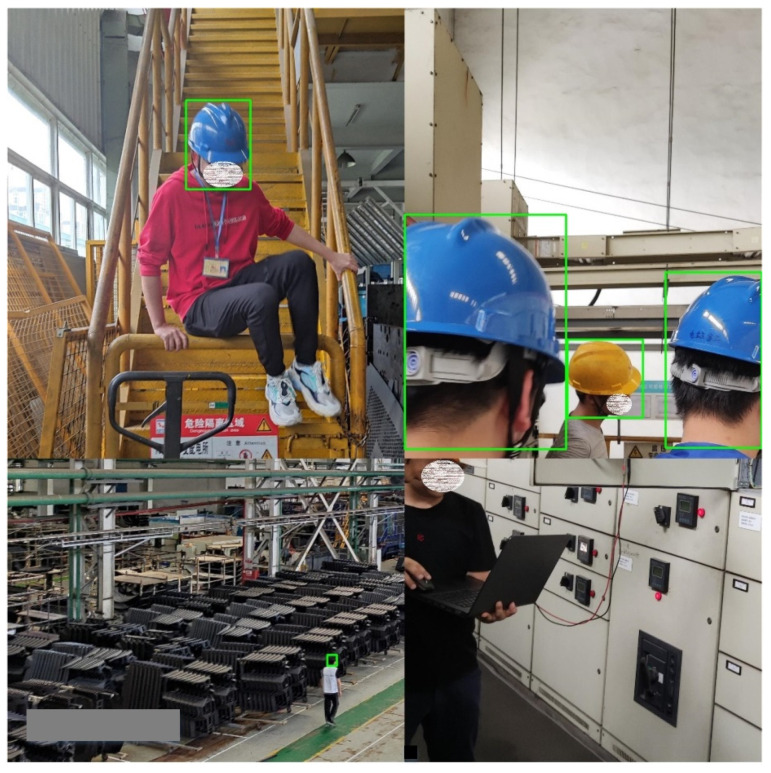
Mosaic data enhancement.

In order to enrich the detection background and increase the number of small objects in the dataset, Mosaic data enhancement was used in the dataset preprocessing stage. By randomly reading four pictures at a time and performing left–right flipping, size scaling, and color gamut changing operations on the four pictures, the four pictures were then placed in the order of upper left, lower left, lower right, and upper right. After that, the fixed area of the picture was cut by a matrix and stitched into a picture, so that a combined picture with a complex background can be obtained, as shown in Figure 10 and Figure 11.

#### 3.5.3. K-Means++ Clustering Algorithm

The K-Means clustering algorithm is introduced in the YOLOv4-tiny model to perform a cluster analysis on each detection object in the dataset, so as to obtain the a priori box that best matches the size of the detection object in the dataset. However, the initial clustering center of the K-Means clustering algorithm is randomly selected, which will affect the final clustering effect. In order to improve the training effect of the model, this paper adopted the K-Means++ clustering algorithm [31] instead of the K-Means clustering algorithm, which can effectively reduce the deviation of the clustering results caused by randomly selecting the initial clustering center. The K-Means++ clustering algorithm flow is as follows:

(1) Randomly select a point from the set of input data points as the initial cluster center.
(2) For each point x in the dataset, calculate its distance D(x) from the nearest cluster center.
(3) Select the second cluster center according to the principle that the larger the point distance D(x) is, the greater the probability of being selected as the cluster center.
(4) Repeat (2) and (3) until the k cluster centers are selected.
(5) Use the above initial cluster centers to perform the standard K-Means algorithm.

**Figure 12:**
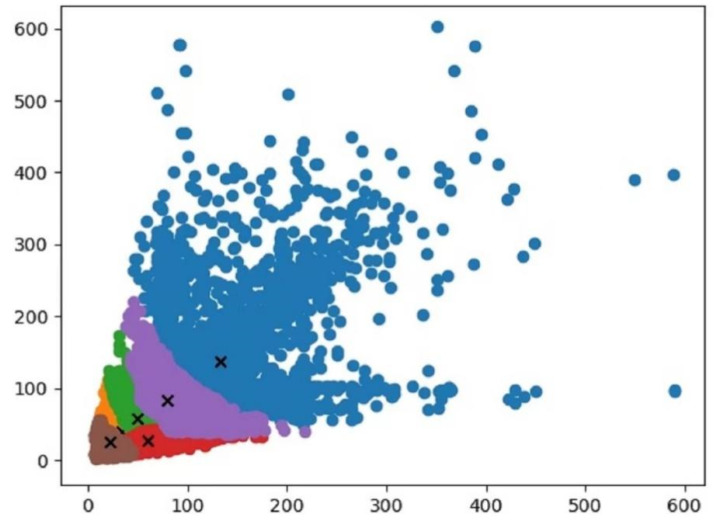
K-Means++ cluster center map. The dots with different colors represent anchor boxes of different sizes. There are six colors in the figure, representing six sizes of anchor boxes. The six symbols “+” represent the cluster centers of the six anchor boxes, respectively.

Among them, the set of input data points is the coordinates of the ground truth box in the dataset, and k is the number of classifications. Since the output feature map of YOLOv4-tiny has 2 scales, the sizes are 26 × 26 and 13 × 13, respectively. Each grid on the feature map of the scale uses three anchor boxes for predictions, thus corresponding to 6 anchor boxes. Since the size of the new anchor box input into the model is more adaptive with the ground truth box size in the dataset, the prediction box generated during the model training process is more accurate, which will help the model to converge quickly. In this study, the six new anchor boxes obtained by the K-Means++ algorithm are: (33, 40), (59, 27), (49, 58), (78, 83), (98, 67), and (133, 137). The cluster center map after K-Means++ processing is shown in Figure 12.

## 4. Experimental Results and Discussion

### 4.1. Experimental Environment Configuration and Training Parameter Settings

This experimental training platform used a desktop computer with the Windows 10 operating system and the main hardware configuration: Intel(R) Xeon(R) CPU @2.40 GHz, NVIDIA GeForce GTX 1050 Ti. The SCM-YOLO algorithm was implemented by the deep learning frameworks Pytorch1.5.1 and python3.6.

The parameters of the model were set as follows: in the first stage, the backbone network was frozen, and the remaining network parameters were trained. The number of samples selected for one training set (batch size) was 64, the dataset was iterated 100 times, and the learning rate was set to 1 × 10^−3^. The second stage unfroze the backbone network and trained all the parameters. The number of samples selected for one training set (batch size) was 32, the dataset was iterated 200 times, and the learning rate was set to 1 × 10^−4^. In order to make the network training process more stable, the idea of migration learning was introduced, and the weight parameters in the already trained CSPDarknet53-tiny network were loaded to speed up the training of the network.

### 4.2. Dataset

**Figure 13:**
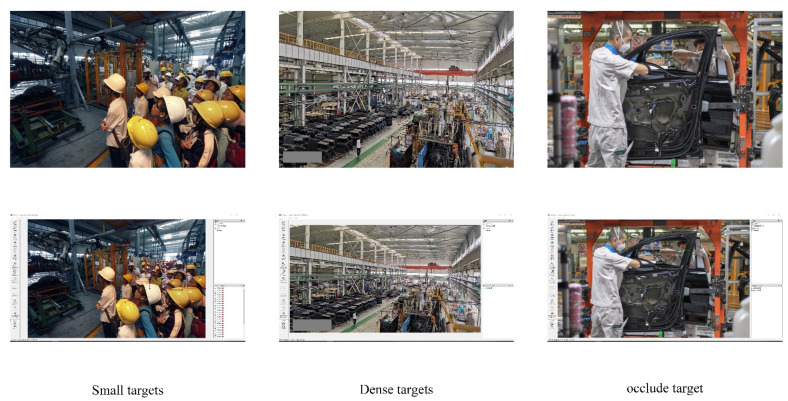
Part of the dataset and LabelMe annotations.

The safety helmet wearing dataset comes from Liuzhou Wuling Automobile Co., Ltd. (Guangxi Automobile Group) and contains 2580 images. Before the experiment, LabelMe was used to manually mark the object. The dataset was then expanded to 11,983 samples using Mosaic data enhancement. The dataset was divided into a training set, validation set, and test set according to the ratio of 6:2:2. The training set included 7189 photos, the verification set included 2397 photos, and the test set included 2397 photos. Part of the dataset images and LabelMe annotations are shown in Figure 13.

### 4.3. Comparative Experiment and Analysis of Results

**Figure 14:**
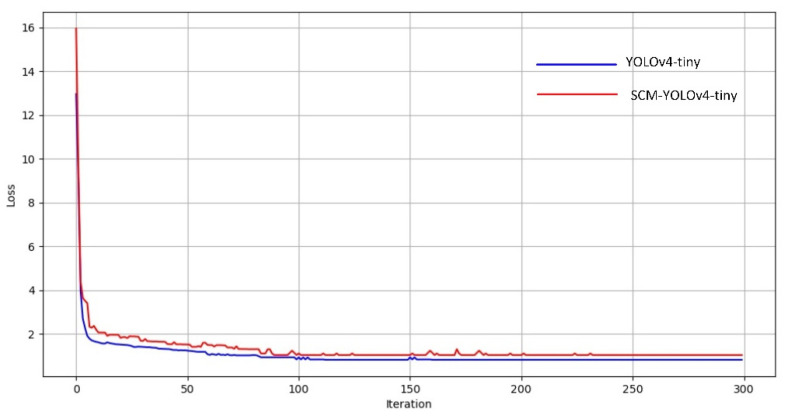
Comparison of the loss functions.

When training the SCM-YOLOv4 model, the change curve of the loss function was drawn through the training results information of each round, as shown in Figure 14. The change of the loss function of the YOLOv4-tiny model was represented by the red line. The change of the loss function of the SCM-YOLOv4 model was represented by the blue line. It can be seen from the figure that the initial loss value of the SCM-YOLO algorithm was smaller than the initial loss value of the YOLOv4-tiny algorithm. After training for 300 times, the loss value of the YOLOv4-tiny algorithm converged to 1, and the loss value of the SCM-YOLO algorithm converged to 0.8.

In order to verify the object detection effectiveness of the algorithm in this paper, comparative tests were performed. The SCM-YOLO algorithm was compared with other object detection models, such as YOLOv3, YOLOv4, YOLOv3-tiny, and YOLOv4-tiny. The same experimental environment and dataset were used for comparative experiments. The Average Precision (AP), Mean Average Precision (mAP), and FPS (detection rate) were calculated and compared, as shown in Table 1.

It can be seen from Table 1 that when the detection speed FPS of the SCM-YOLO algorithm met the actual application requirements, the mAP reached 93.1%, which was 4.7 percentage points higher than that of the YOLOv4-tiny algorithm. Among them, the AP of head and safety helmet detection increased by 5.8% and 3.7%, respectively, but the detection speed decreased slightly. Compared with the YOLOv3 algorithm, the mAP was increased by 0.6%, and the detection speed was significantly improved. Compared with the YOLOv3-tiny algorithm, the mAP was increased by 13.2 percentage points, and the detection speed was also slightly improved. Compared with YOLOv3 and YOLOv3-tiny, mAP was increased by 0.6% and 13.2%, respectively. Compared with the YOLOv4 and YOLOv5 algorithms, the SCM-YOLO algorithm has almost three times the detection speed of the YOLOv4 and YOLOv5 algorithms when the mAP is not much different. As a relatively mature algorithm in the YOLO series of algorithms, the YOLOv5 algorithm has excellent detection accuracy. In this experiment, mAP can reach 96.7%. However, as far as the practical application scenarios of this study are concerned, the detection speed cannot meet the application requirements of real-time detection. However, on the whole, the SCM-YOLO algorithm proposed in this paper has a good comprehensive performance in detection accuracy and speed, which was enough to meet the task requirements in various complex scenarios.

**Figure 15:**
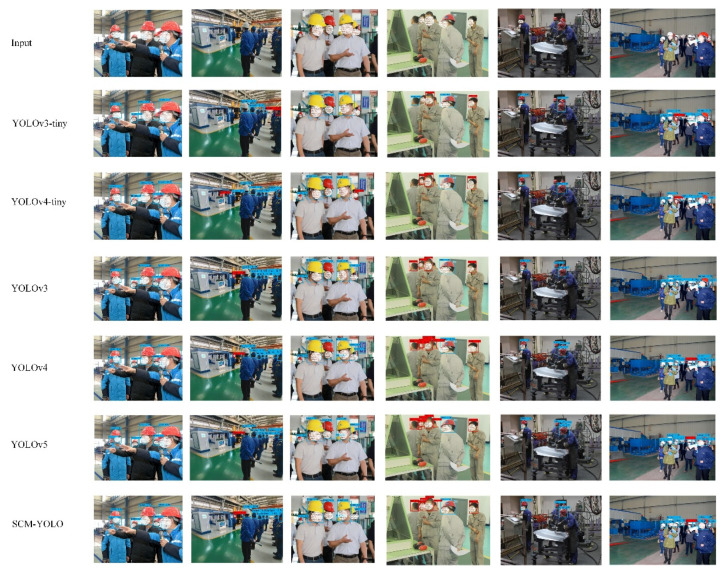
Comparative experimental results.

In order to more intuitively verify the object detection effect and model robustness of the SCM-YOLOV4 algorithm in different complex scenarios, this paper selected the same test dataset for the experimental comparison of YOLOV3-tiny, YOLOv3, YOLOV4-tiny, YOLOv4, YOLOv5, and SCM-YOLO. The experimental results are shown in Figure 15. There are a total of six targets in the fourth test image, including one positive sample (with helmet) and five negative samples (without helmet). The YOLOv3-tiny algorithm detected a total of four targets, two missed detections, and one false detection. The YOLOv4-tiny algorithm detected a total of four targets, two missed detections, and zero false detections. The YOLOv3 algorithm detected a total of five targets, one missed detection, and zero false detections. The YOLOv4, YOLOv5, and SCM-YOLO algorithms correctly detected all the targets. In the sixth test image, the detected target scene is a dense scene. The YOLOv3-tiny algorithm and the YOLOv4-tiny algorithm had serious missed detections and false detections, and the YOLOv3 algorithm had serious missed detections. Although the YOLOv4, YOLOv5, and SCM-YOLO algorithms had a small number of missed detections, the overall detection effect was excellent. It can be seen from the above that, in the YOLOv3-tiny algorithm, there were serious false detections and missed detections for small objects and occluded objects. The YOLOv3 and YOLOv4-tiny algorithms improved the detection effect of occluded objects, but the problem of missed detection was still serious compared with the YOLOv3-tiny algorithm. Among them, the YOLOv4, YOLOv5, and SCM-YOLO algorithms had excellent performances in all aspects of the test set, and the detection accuracy of small objects and occluded objects was relatively high.

### 4.4. Ablation Experiment and Analysis of Results

**Figure 16:**
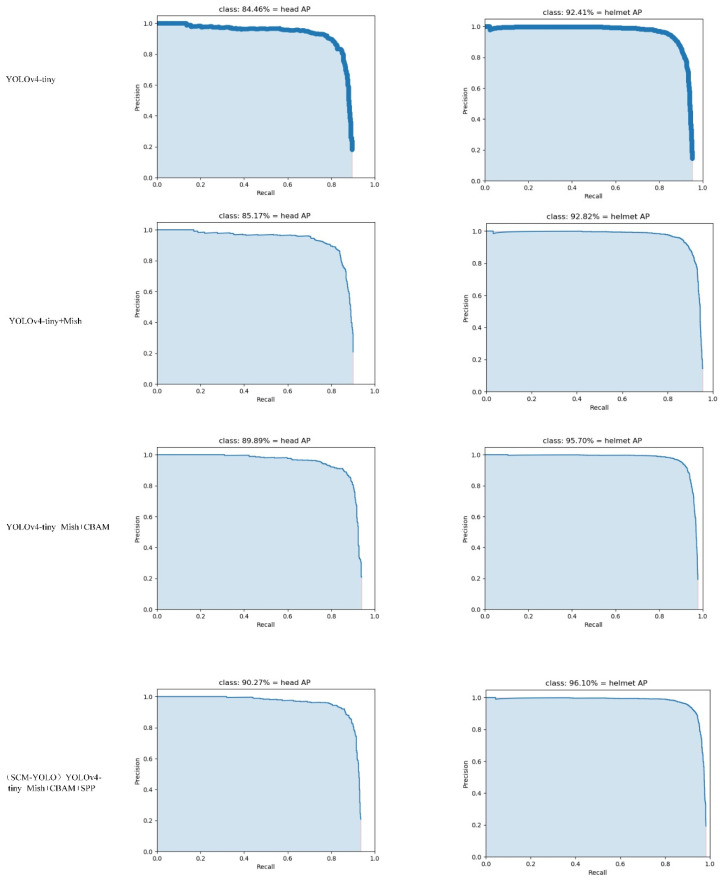
AP curves for head and helmet.

We conducted ablation experiments to explore the effects of the Mish activation function, SPP module, and attention mechanism on the model performance. The results of the ablation experiment are shown in Table 2. Four groups of models were trained, respectively, and each module was successively added on the basis of YOLOV4-tiny. Four groups of models were tested in turn on the same test set, and the AP curves of head and helmet detection are shown in Figure 16. Compared with the results of yOLOV4-tiny and YoloV4-tiny+Mish, the AP value and mAP value of the yOLOV4-tiny+Mish model were slightly improved. The backbone network with the Mish activation function optimized the feature extraction ability of the model and improved the detection performance. Compared with the YOLOv4-tiny+Mish+CBAM model, the mAP of the latter was increased by 4.36%, and the AP values were increased by 5.43% and 3.29%, respectively. It can be found that adding the CBAM attention mechanism effectively optimized the model performance and improved the detection accuracy of the model by increasing the weight of the region of interest. Compared with the results of YOLOv4-tiny+Mish+CBAM and YOLOv4-tiny+Mish+CBAM+SPP, it can be found that the proposed model combined with the improved SPP structure had a slight improvement in AP and mAP. The extraction of effective feature information and multiscale feature fusion enabled the model to better detect safety helmet wearing objects. On the whole, each module in the SCM-YOLO algorithm proposed in this paper effectively improved the detection accuracy, met the actual detection requirements, and verified the feasibility of the model.

### 4.5. CBAM Visualization Experiment

**Figure 17:**
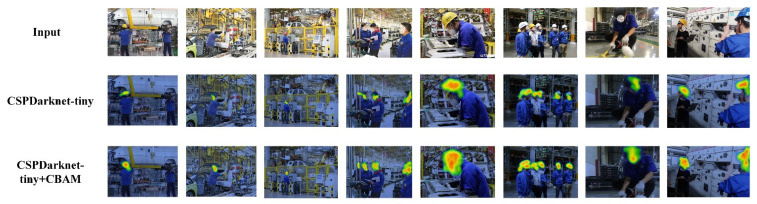
CBAM visualization experiment results.

It can be seen from Table 2 that the model with the CBAM attention mechanism had a higher detection accuracy than the other models. In order to explore the specific impact of CBAM on the model, this paper used Grad_CAM (Gradient-weighted Class Activation Mapping) to visualize the performance of the CSPDarknet53-tiny network and the CSPDark-net53 network with the CBAM attention mechanism. The results are shown in Figure 17.

As can be seen from Figure 15, for the original CSPDarknet53-tiny network, the extracted features were obvious features on the object that did not completely cover the object and were prone to false detection and missed detection. The network with CBAM not only extracted the main features but also gave higher weight information to the secondary features. Therefore, the feature information extracted by the proposed network for the target object is more abundant. The proposed model can achieve the more accurate detection of targets and reduce false detection and missed detection.

## 5. Conclusions

Aiming at the problem of the low detection accuracy of safety helmet wearing in the production workshop, this paper proposed a real-time detection model for workshop safety helmet wearing based on the SCM-YOLO model. Based on YOLOv4-tiny, we changed the activation function to smooth nonmonotonic activation function Mish, introduced the SPP structure, and added the CBAM module. At the same time, the k-means clustering algorithm was optimized to the K-Means ++ clustering algorithm so as to improve the accuracy, robustness, and generalization ability of the model. The experimental results showed that the convergence value of the loss function of the SCM-YOLO algorithm was smaller. Compared with the YOLOv4-tiny algorithm, the mAP reached 93.19%, an increase of 4.76%, and its detection speed of 22.9 FPS was sufficient for practical applications. Therefore, the SCM-YOLO algorithm is more feasible than the YOLOv4-tiny algorithm.

Although the SCM-YOLO algorithm proposed in this paper can better solve the problem of low accuracy of safety helmet wearing detection, there are still many optimizable issues to be further perfected in the future. For example, in the comparative experiment, the SCM-YOLO algorithm still has a small number of missed detections when the detection targets are too dense. For such special cases, the number of dense target datasets can be increased in future experiments, and the model performance would be better by introducing more effective attention mechanisms or backbone networks. This study verifies the feasibility of the model, but experiments for special cases still need to be improved.

## Figures and Tables

**Table 1:** Performance comparison of object detection algorithms.

Algorithm	FPS/s^−1^	AP/%		mAP/%
		Head	Helmet	
YOLOv3	8.5	92.3%	92.7%	92.5%
YOLOv4	8.1	95.1%	97.5%	96.3%
YOLOv3-tiny	19.4	76.2%	83.6%	79.9%
YOLOv4-tiny	24.1	84.4%	92.4%	88.4%
YOLOv5	8.4	96.1%	97.3%	96.7%
SCM-YOLO	22.9	90.2%	96.1%	93.1%

**Table 2:** Ablation experiment results.

Model	AP/%		mAP/%
	Head	Helmet	
YOLOv4-tiny	84.46%	92.41%	88.43%
YOLOv4-tiny+Mish	85.17%	92.82%	88.99%
YOLOv4-tiny+Mish+CBAM	89.89%	95.70%	92.79%
(SCM-YOLO) YOLOv4-tiny+Mish+CBAM+SPP	90.27%	96.10%	93.19%

## Data Availability

The safety helmet wearing dataset comes from Liuzhou Wuling Automobile Co., Ltd. (Guangxi Automobile Group). Since the dataset involves company privacy issues, the dataset is not open to the public. All results obtained from this dataset in this paper have been approved for use.
